# The Global Fund in China: Multidrug-resistant tuberculosis nationwide programmatic scale-up and challenges to transition to full country ownership

**DOI:** 10.1371/journal.pone.0177536

**Published:** 2017-06-19

**Authors:** Lixia Wang, Renzhong Li, Caihong Xu, Hui Zhang, Yunzhou Ruan, Mingting Chen, Dongmei Wang, Emilio Dirlikov, Xin Du, Jin Zhao, Yanlin Zhao, ShengFen Wang, Yuhong Liu, Liang Li, Dennis Falzon, Yanni Sun, Yu Wang, Bernhard Schwartländer, Fabio Scano

**Affiliations:** 1 National Center for TB Control and Prevention, Chinese Center for Disease Prevention and Control, Beijing, China; 2 Department of Anthropology, McGill University, Montréal, Québec, Canada; 3 Beijing Chest Hospital, Clinical Centre on Tuberculosis, Chinese Centre for Disease Prevention and Control, Beijing, China; 4 Global TB Programme, World Health Organization, Geneva, Switzerland; 5 China Country Office, World Health Organization, Beijing, China; Johns Hopkins University Bloomberg School of Public Health, UNITED STATES

## Abstract

China has the world’s second largest burden of multidrug-resistant tuberculosis (MDR-TB; resistance to at least isoniazid and rifampicin), with an estimated 57,000 cases (range, 48,000–67,000) among notified pulmonary TB patients in 2015. During October 1, 2006–June 30, 2014, China expanded MDR-TB care through a partnership with the Global Fund to Fight AIDS, Tuberculosis, and Malaria (Global Fund). We analyzed data on site expansion, patient enrolment, treatment outcomes, cost per patient, and overall programme expenditure. China expanded MDR-TB diagnostic and treatment services from 2 prefectures in 2006 to 92 prefectures, covering 921 of the country’s 3,000 counties by June 2014. A total of 130,910 patients were tested for MDR-TB, resulting in 13,744 laboratory-confirmed cases, and 9,183 patients started on MDR-TB treatment. Treatment success was 48.4% (2011 cohort). The partnership between China and the Global Fund resulted in enormous gains. However, changes to health system TB delivery and financing coincided with the completion of the Global Fund Programme, and could potentially impact TB and MDR-TB control. Transition to full country financial ownership is proving difficult, with a decline in enrollment and insufficient financial coverage. Given needed improvement to the current treatment success rates, these factors jeopardise investments made for MDR-TB control and care. China now has a chance to cement its status in TB control by strengthening future financing and ensuring ongoing commitment to quality service delivery.

## Introduction

Drug resistant forms of tuberculosis (TB) pose a serious threat to control efforts. Globally, in 2015 there were an estimated 580,000 (range, 520,000–640,000) new cases of multidrug-resistant, defined as resistance to at least isoniazid and rifampicin, or rifampicin-resistant TB (MDR/RR–TB) [1]. Drug-resistance among TB patients varies geographically, with the highest reported proportions occurring in Eastern Europe and Central Asia, while India and China are estimated to have the first and second largest burdens of MDR-TB respectively [1,2].

In 2007, China conducted its first national TB drug-resistance survey, finding that 5.7% (range, 4.5–7.0%) of new and 25.6% (range, 21.5–29.8%) of retreatment TB patients were MDR-TB [3]. For 2015, WHO estimates the total number of MDR/RR-TB patients among notified cases of pulmonary TB in China was 57,000 (range, 48,000–67,000) cases. That year, of 9,662 laboratory-confirmed patients with MDR/RR-TB, 5,691 (59%) were started on treatment; of 357 laboratory-confirmed patients with extensively drug-resistant TB (XDR-TB), defined as MDR-TB plus additional resistance to a fluoroquinolone and a second-line injectable, 122 (34%) patients were started on treatment [1].

In 2003, China started a partnership with the Global Fund to Fight AIDS, Tuberculosis, and Malaria (Global Fund) on TB control. Under the partnership, China initiated programmes targeting MDR-TB in October 2006, and continued programme activities until Global Fund support ended on June 30, 2014.

In this article, we review China’s MDR-TB scale-up initiated in collaboration with the Global Fund, from October 2006 to June 2014 inclusive. We describe programmatic scale-up, focused on: site expansion, patient enrolment, treatment outcomes, cost per patient, and programme expenditure. We conclude with a discussion of national health reforms that assumed responsibility for MDR-TB services.

## Patients and methods

### Patients

**Fig 1:**
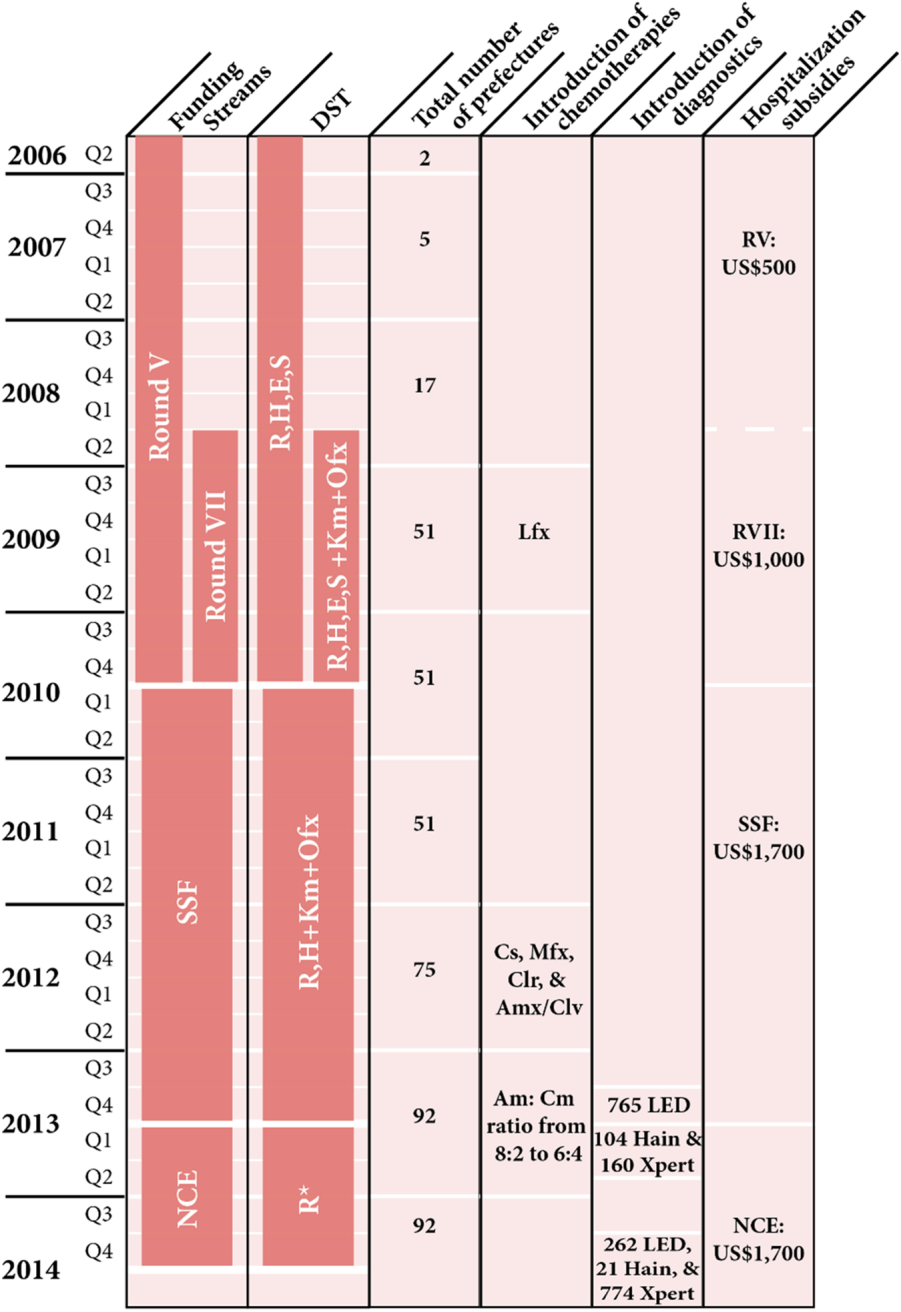
Timeline schematic of China Global Fund MDR-TB programme scale-up. Quarters (Q) reflect the funding streams and not calendar year. * DST for at least R under NCE only available at selected sites using Xpert, where resistance for R was used as a proxy for MDR-TB. Culture-based DST was run in parallel to adjust treatment options in case of resistance. MDR-TB Regime: Intensive Phase: Z, Km (Am, Cm), Lfx (Mfx), Cs* (PAS, E), Pto; Continuation Phase: Z, Lfx (Mfx), Cs* (PAS, E), Pto. XDR-TB Regime: Intensive Phase: Z, Cm, Mfx, PAS, Cs*, Pto, Clr, Amx/Clv; Continuation Phase: Z, Mfx, PAS, Cs*, Pto, Clr, Amx/Clv. Drug Acronyms: Am = Amikacin, Amx/Clv = Amoxicillin plus clavulanate; Cm = Capreomycin, Clr = Clarithromycin, Cs = Cycloserine, E = Ethambutol, Km = Kanamycin, Lfx = Levofloxacin, Mfx = Moxifloxacin, PAS = p-aminosalicylic acid, Pto = Prothionamide, Z = Pyrazinamide. Drug names in parentheses represent acceptible replacements. Acronyms: DST = Drug-sensitivity testing; Hain = MTBDR(plus) assay machines; LED = LED microscopes; NCE = No cost extension; RV = Round V; RVII = Round VII; SSF = Single-stream funding; Xpert = Xpert MTB/RIF (Cepheid).

Testing for MDR-TB commenced in October 2006 among high-risk populations, as per WHO classification [4], under Round V (RV) and Round VII (RVII) funding (Fig 1). On July 1, 2010, disparate funding streams were consolidated into a single-stream funding (SSF) mechanism. In June 2013, a no cost extension (NCE) was approved for 1 year, and Global Fund support ended on June 30, 2014.

**Fig 2:**
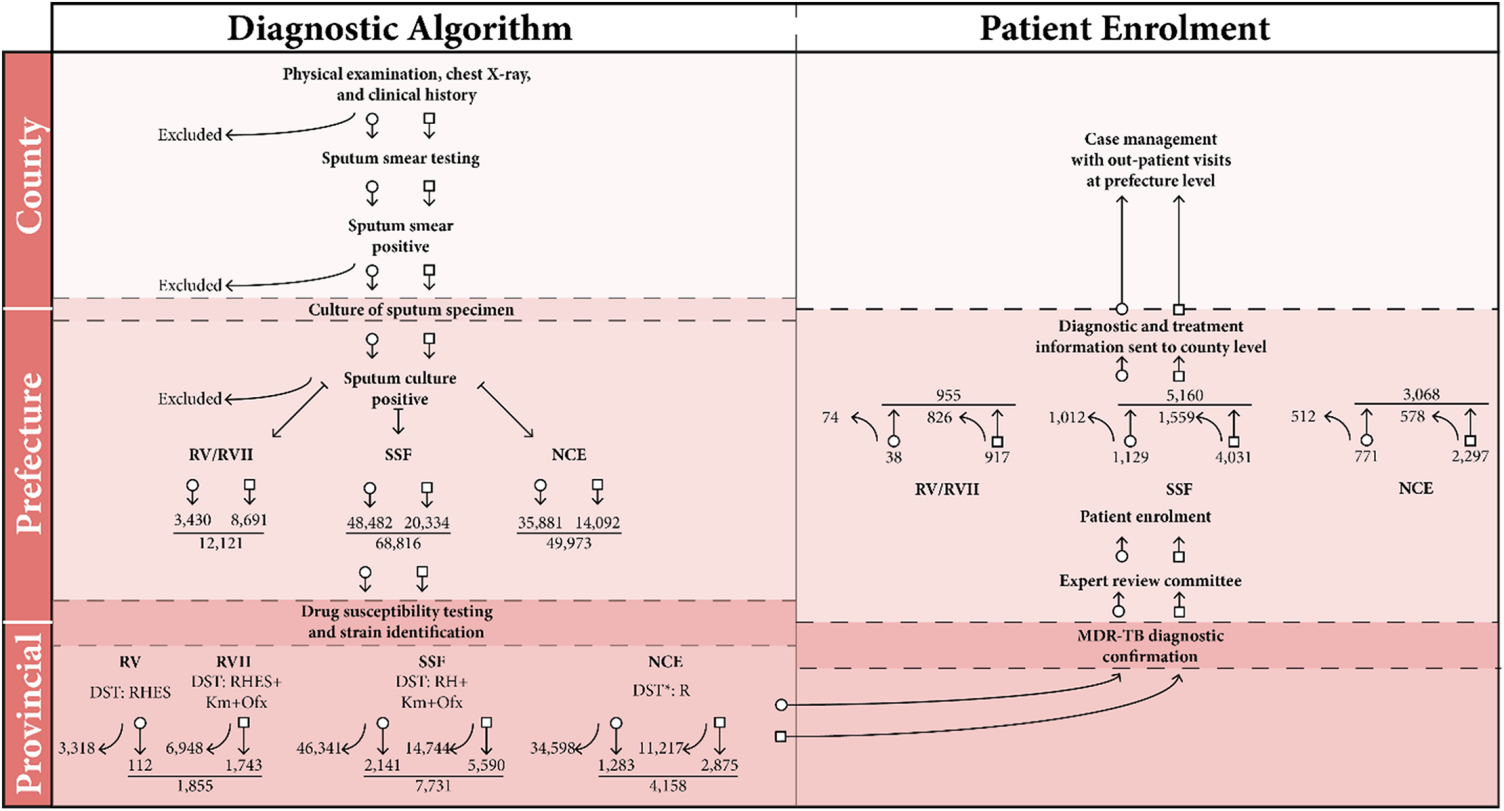
Diagnostic algorithm and patient enrolment by administrative level—China, 2006–2013. DST = Drug sensitivity testing; E = Ethambutol; H = Isoniazid; Km = Kanamycin; MDR-TB = Multridrug-resistant tuberculosis; NCE = No cost extension funding, July 1, 2013 to June 30, 2014; Ofx = Ofloxacin; R = rifampicin; RV = Round V funding, October 1, 2006 to June 30, 2010; RVII = Round VII funding, October 1, 2008 to June 30, 2010; S = Streptomycin; SSF = Single-stream funding, July 1, 2010 to June 30, 2013. Open circles represent continuation along the algorithm for new cases, while open boxes represent continuation for other high risk groups. Curved lines represent exclusion from the programme. * Drug sensitivity testing for at least rifampicin (R) under NCE only available at sites using Xpert.

Patients with presumptive MDR-TB or XDR-TB, defined as MDR-TB plus additional resistance to a fluoroquinolone and a second-line injectable, were identified using electronic TB-specific reporting mechanisms. China’s national TB programme (NTP) is based on an administrative hierarchy, organised sub-nationally at three descending levels: province, prefecture, and county. As per national policy, risk factors include: failure to respond to initial or retreatment efforts, chronic cases of TB, close contact with a known MDR-TB patient, smear positive cases at the end of 2 to 3 months of initial treatment [5,6]. Patients were screened for MDR-TB according to the project’s diagnostic algorithm (Fig 2).

Patients presented at county-level health institutions, where sputum testing was conducted and drug-resistance risk assessment was initiated. Specimens were collected and tested within 3 days. Sputum samples from cases at risk for MDR-TB were referred to the county, prefecture or provincial-level laboratory for culture testing. For samples with a positive culture result, strain identification and drug-susceptibility testing (DST) (agar-proportion method) were conducted at prefecture-level laboratory or provincial-level reference laboratory.

Throughout the program, DST adapted in line with global policies changes as well as improved programmatic capacity to test for second-line TB drugs and correlate results to treatment options (Fig 2) [7]. In May 2013, 765 light emitting diode (LED) microscopes were introduced, and in April 2014 an additional 262 LED microscopes were added. In July 2013, 104 Genotype MTBDRplus assay (Hain Lifescience) machines were introduced, with an additional 21 machines added in July 2014. In July 2013, 160 Xpert MTB/RIF (Xpert; Cepheid) machines were introduced, with an additional 774 machines purchased in July 2014.

Upon diagnostic confirmation, patients were officially enrolled into the Global Fund project through a prefecture-level expert review committee. All patients provided informed consent before treatment enrolment. Based on DST results, most MDR-TB patients received a standardized treatment regimen, consisting of a 6-month intensive phase (8 months if sputum positive at 6 months), followed by an 18-month continuation phase. For XDR-TB cases, the standardized regimen consisted of a 12-month intensive phase, followed by an 18-month continuation phase. Following principles of TB clinical practice, effective four-drug regimens for individualized treatment were tailored according to DST results and patients’ needs, including cases of further drug resistance, pregnancy, breastfeeding, diabetes, and children (i.e., ≤15 years of age).

Prior to initiating treatment, medical histories were collected, and patients underwent a thorough physical examination. Patients commenced treatment through hospitalization for up to 2 months. Patients were treated on an out-patient basis, with direct observation of treatment conducted by trained staff at appropriate county-level health institutions closest to patients’ homes. Routine follow-up examinations and laboratory tests were conducted every month during the intensive phase, and every 2 months during the continuation phase.

Following WHO definitions [8], treatment outcomes were defined as follows: 1) cured, 2) treatment completed, 3) died, 4) treatment failed, 5) lost to follow-up, and 6) not evaluated.

### Data collection

Data were collected by each project site and reported to the Global Fund provincial-level project offices, which conducted data quality assessment and reconciled discrepancies. Data were then reported to the Global Fund’s national project office in Beijing for final assessment. During RV, RVII, and SSF, patient data were systematically reported to and stored at the national project office through a paper-based quarterly system. Under NCE, this system was progressively replaced by an electronic reporting and recording system. Data on cost per patient, programme expenditure, and the deployment of new diagnostic machines were sourced from the Beijing offices.

### Statistical analysis

All data were analyzed anonymously. Aggregated figures at the provincial level based on the data sources were used. Data were pooled across provinces and years to express global values in absolute numbers and percentages. Statistics were generated using R, and the choropleth maps and several figures were created using the *ggplot2* package working in the same environment (version 3.0.1) [9,10]. The maps were plotted using shape files for the first administrative level downloaded from GADM [11].

## Results

### Site expansion

The MDR-TB component of the NTP began on October 1, 2006 with sites in two prefectures: Shenzhen (Guangdong Province) and Wuhan (Hubei Province). By June 30, 2014, the geographic coverage had expanded to sites in 30 of China’s 31 provinces, representing 92 (28%) of 333 total prefectures, covering 921 (28%) of 3,300 counties (S1 Fig).

### Case detection and patient enrolment

**Fig 3:**
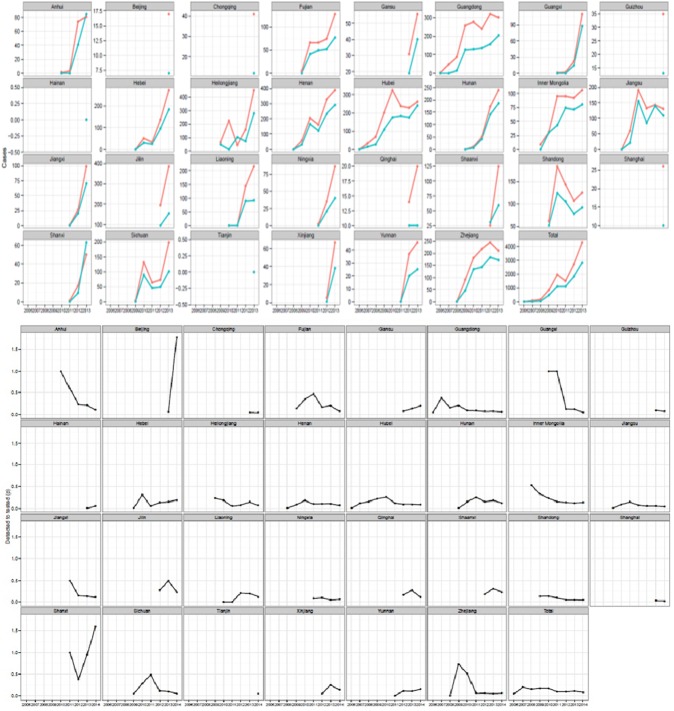
MDR-TB cases detected (pink) compared with TB cases enrolled on MDR-TB treatment (blue), and ratios of MDR-TB cases detected to tested, by province—China, October 2006–June 2014.

During October 1–December 31, 2006, no patients were enrolled for treatment. At the provincial level, case detection of MDR-TB and patient enrolment increased from 2006 to 2013, under RV, RVII, or SSF (Fig 3). In 2011, both case detection and enrolment decreased due to a temporary “freeze” of Global Fund support between May and September (Fig 3). In general, at the provincial level, case detection increased over time, while the ratio of cases of MDR-TB detected to patients enrolled remained stable (Fig 3).

By the programme’s completion, 130,910 presumptive TB patients were evaluated, leading to 13,744 diagnostic confirmations of MDR-TB and 9,183 patients enrolled on treatment (Table 1). Among these, seven pediatric MDR-TB patients were enrolled. Between 2012 and June 2014, out of 5,031 patients enrolled in the MDR-TB programme, 351 were XDR-TB. Under NCE, the project expanded rapidly: in one year, 49,973 presumptive cases were tested, leading to 4,158 diagnostically-confirmed cases, and 3,068 patients enrolled. This constitutes 38.2%, 30.3%, and 33.4% of the presumptive, diagnostically-confirmed, and enrolled patients under all funding rounds combined, respectively.

**Table 1:** Patient breakdown.

	Presumptive Cases			Diagnosis			Enrolled for treatment		
	RV & RVII	SSF	NCE	RV & RVII	SSF	NCE	RV & RVII	SSF	NCE
**New cases**	3,430	48,482	35,881	112	2,141	1,283	38	1,129	771
**Other high risk groups**	8,691	20,334	14,092	1,743	5,590	2,875	917	4,031	2,297
**Funding round** **total (%)**^**a**^	12,121 (9)	68,816 (53)	49,973 (38)	1,855 (14)	7,731 (56)	4,158 (30)	955 (10)	5,160 (56)	3,068 (34)
**Cumulative** **Total**	130,910			13,744			9,183		

Round V (RV) funding began on October 1, 2007 and ended on June 30, 2010, and Round VII (RVII) began on October 1, 2008 and ended on June 30, 2010. Single-stream funding (SSF) began on July 1, 2010 and ended on June 30, 2013. The no cost extension (NCE) period began on July 1, 2013 and ended on June 30, 2014.

^a^ Percentage from cumulative total.

### Treatment outcomes

**Fig 4:**
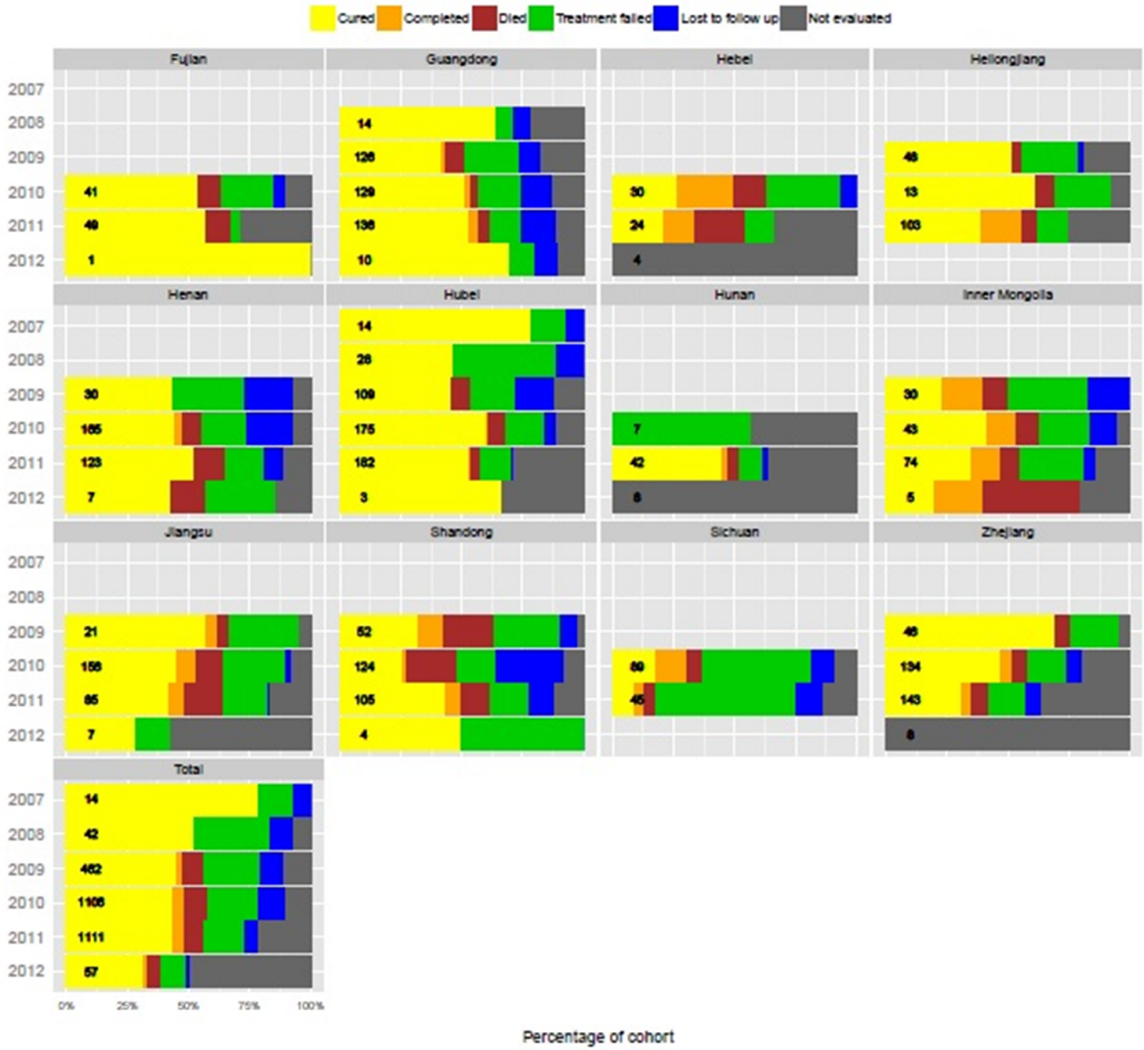
Treatment outcomes for patients diagnosed with MDR-TB by province, 2007 to 2012 cohorts. Total number of cases in each annual cohort shown in bars.

Of the 6,115 patients enrolled for treatment under RV, RVII, and SSF funding, 5,858 (96%) completed 6 months of treatment. Treatment outcomes varied by province and year, with treatment success rate remaining stable or declining in most provinces (Fig 4). During 2009–2011, treatment success improved in provinces, like Guangdong and Hubei, with reductions in treatment failure, loss to follow up, and deaths; in other provinces, like Jiangsu, the treatment success rate declined as a result of increasing mortality. In a number of provinces (e.g., Hebei, Heilongjiang, Hubei and Zhejiang) treatment success declined, in part due to cases reported as “not evaluated.”

Treatment outcomes were available for 2,792 patients treated until 2012, of which, 1,226 (43.9%) patients were bacteriologically-confirmed as cured, and 124 (4.4%) completed treatment without bacteriological cure; the overall treatment success rate was 48.4%. Differences by province were observed (S1 Table), with the highest treatment success rate in Fujian Province (56%) and the lowest in Sichuan Province (24.6%). Patients lost to follow-up or not evaluated comprised 8.4% and 9.5% of patient treatment outcomes, respectively; 543 (19.4%) patients sustained a treatment failure, of which 192 (35%) patients failed due to adverse drug reactions. Advanced or complicated cases led to poor treatment outcomes in Henan, Jiangsu, Shandong, Sichuan, and Zhejiang provinces.

There was a positive correlation between sputum smear and culture conversion at 6 months (S2 Fig). As expected from the operating characteristics of the two tests, most provinces reported higher levels of sputum smear conversion than culture. However, several notable outliers were observed where conversion levels were equivalent (Hebei) or even higher when compared with culture (Yunnan and Qinghai). The departure from the trend lines was not related to the number of patients receiving MDR-TB treatment in a given province.

### Cost per patient

**Fig 5:**
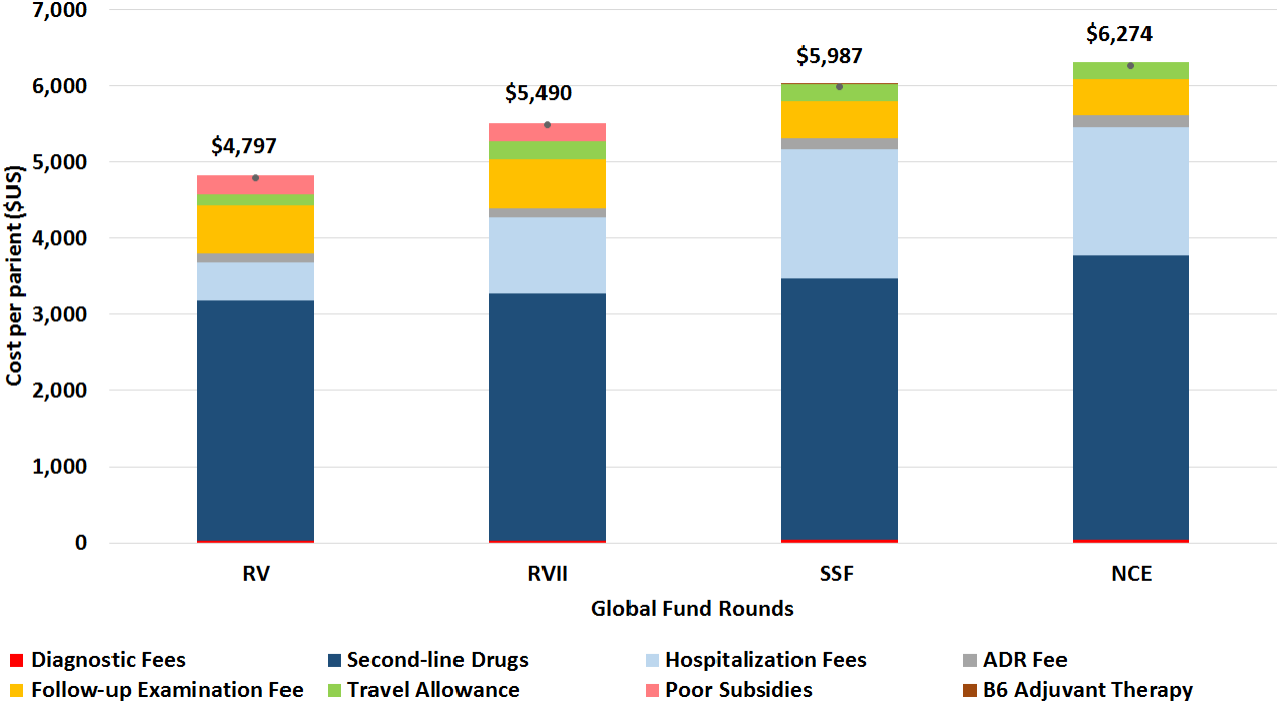
Cost per patient by line item, under four Global Fund funding rounds. Round V (RV) funding began on October 1, 2007 and ended on June 30, 2010; Round VII (RVII) began on October 1, 2008 and ended on June 30, 2010; Single-stream funding (SSF) began on July 1, 2010 and ended on June 30, 2013; the no cost extension (NCE) period began on July 1, 2013 and ended on June 30, 2014. ADR Fee refers to “adverse drug reaction fee.” Under SSF, vitamin B6 adjuvant therapy was also provided.

Patient costs ranged from USD 4,797 during RV to USD 6,274 during NCE (Fig 5). Under each Global Fund round, the cost per patient differed by composition of expenditures and total cost. Hospitalization fees account for much of these differences, at US$500 under RV, US$1,000 under RVII, and US$1,700 under both SSF and NCE, representing 10.4%, 18.2%, 28.4%, and 27.1% of the total cost per patient for RV, RVII, SSF, and NCE, respectively. In RV, only 20% of patients were hospitalised, and increased hospitalization provisions were put in place to more adequately cover patient expenses. Second-line drugs accounted for more than 50% of overall costs during all four phases. Under RV, standardized second-line treatment cost US$3,153, increasing to US$3,250 under RVII due to the addition of levofloxacin (Lfx) in 2009. Under SSF, cycloserine (Cs), moxifloxacin (Mfx), clarithromycin (Clr), and amoxicillin plus clavulanate (Amx/Clv) were added in 2012, with total treatment cost of US$3,432. In 2013 under NCE, cost increased to US$3,727 in order to regulate the amikacin (Am): capreomycin (Cm) ratio from 8:2 to 6:4. Under SSF, the Chinese Ministry of Civil Affairs took over responsibility for providing subsidies for poor patients.

### Programme expenditure

**Fig 6:**
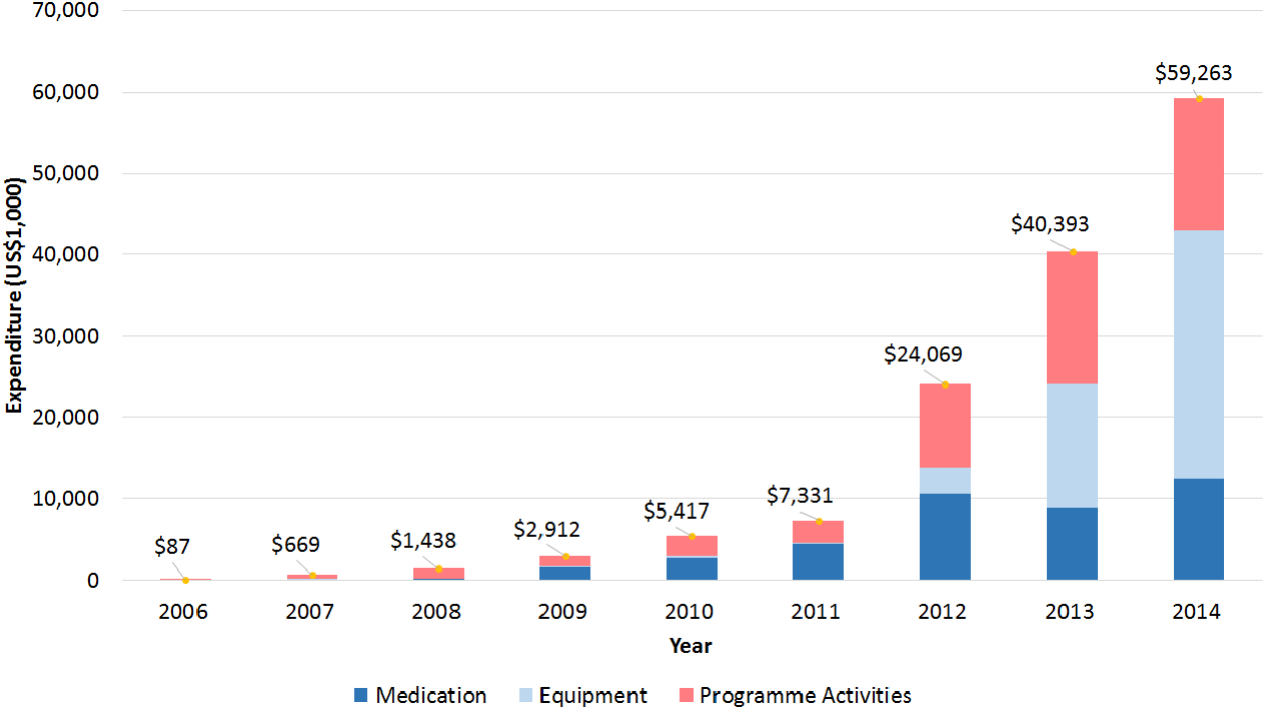
Programme expenditure, by year, and disaggregated by medication, equipment, and activities (in US$1,000). The Global Fund programme operated only for 3 months (October–December) in 2006, and 6 months (January–June) in 2014.

Total programme expenditure increased throughout implementation, from US$86,748 during October–December 2006, to over US$59.2 million for the first 6 months of 2014 (Fig 6). Expenditure was allocated by three areas: medication, equipment, and programme activities (e.g., training, meetings, supervision, patient costs, and human resources). In 2006, the entire budget was dedicated to programme activities; in 2014, programme activities accounted for over US$16.2 million (27%). Equipment was first purchased in 2007, totaling US$242,467; in 2014, equipment costs accounted for over US$30.5 million. Medications accounted for 13.9% of total expenditure in 2008, the first year medications were introduced into the programme, and grew to 21% of the total expenditure in 2014. The overall combined programme expenditure was over US$141.5 million, distributed as follows: 29% for medication, 35% for equipment, and 36% for programme activities.

## Discussion

Since October 2006, China quickly scaled-up its MDR-TB programme in collaboration with the Global Fund. This included: an increased number of sites, expanding from two to 92 (28%) of China’s 333 total prefectures, covering 921 (28%) of 3,300 counties; strengthened capacity to rapidly diagnose drug sensitivity through the roll-out of LED microscopes, Genotype MTBDR(plus) assays, and Xpert MTB/RIFGeneXpert machines; and the ability to provide patients with appropriate treatment regimens. While the scale-up is impressive, challenges remain.

First, despite a 2010 policy to increase drug-susceptibility testing among new TB cases, the programme experienced major difficulties identifying and correctly diagnosing patients at risk of MDR-TB. Given the limited number of patients that benefited from the piloting of rapid diagnostics initiated during the final period of the Global Fund support, analysis could not determine how rapid diagnostics affected reliability and timeliness, or treatment outcomes. Access to MDR-TB diagnosis and treatment remain a challenge for children who were only marginally included in this program, and future efforts should prioritize improving access to MDR-TB diagnosis and treatment services for children.

Second, the overall treatment success rate was 48.4%, approximating the global average for MDR-TB patients in the 2011, which was 48% [1]. For MDR-TB patients treated through the program, treatment cohorts from 2009–2011 are more relevant for interpretation, given that 2007–2008 cohorts were too small, and many cases in the 2012 cohort have not been evaluated. The 2009–2011 cohorts indicate a negligible variance in terms of treatment success rates, but highlight key inter-provincial differences. As the only province in the Western Region with treatment outcomes, Sichuan Province had the lowest treatment success rate. Special attention should be given to the Western Region, where the burden of drug-susceptible TB is higher [12], and financial and hospital-based TB resources are scarcer [13]. Achieving good treatment outcomes remains the main goal of a functioning programme. The introduction of additional drugs in 2012, in particular CS, may have resulted in improved treatment outcomes of subsequent cohorts (Fig 1). Programmatic challenges and inequalities across the provinces provide a clue to low treatment success rates. Future analysis comparing patients who achieved favorable treatment outcomes with those who did not will inform how treatment outcomes can be improved, as well as whether DST results correlate to treatment outcomes.

Third, MDR-TB centres are mostly located at urban hospitals. Decentralizing care to lower administrative levels will rely heavily on semi- and untrained doctors. While bringing care closer to the patients will likely improve compliance and lower overall costs, it also presents difficulties for maintaining quality of care.

Finally, although costs for MDR-TB care remain high, the benefits of the investment in terms of lives saved and additional cases of MDR-TB prevented well outweigh the current cost of care. Under NCE, the cost per patient was US$6,274, slightly below China’s 2013 GDP per capita estimated at US$6,807 [14]. As elsewhere [15], TB patients in China are generally poor, with 82% of TB patients having household income below the national average [16,17], and patients with drug-resistant forms are no exception [18,19]. Future efforts should monitor patients' out-of-pocket expenditures to ensure that programs provide sufficiently coverage of medical expenditures.

In addition to these challenges, on-going health reforms in China coincide with the completion of the Global Fund programme. Changes to health system delivery and financing will potentially impact TB and MDR-TB control. TB diagnostic and treatment services are shifting from services delivered by CDCs to specialized public hospitals. As such, public health hospitals will be at the core of the TB continuum of care and control. Furthermore, earmarked funds are being replaced by innovative mixed-source public health funding pools, combining central and provincial level funds, health insurance schemes, and out-of-pocket patient expenditure.

The challenge is to ensure that quality services will improve from current treatment outcome rates during this transition, which is happening within a comparatively short period of time. New policies, clear regulations, and close monitoring are crucial to ensure service delivery follows international standards, and to avoid increased patient expenditure, higher hospitalization rates, and the wasteful delivery of clinical services [20,21].

For MDR-TB, the concomitant completion of the Global Fund project and on-going health reforms poses significant challenges. In 2014, the Global Fund provided 26% of the total reported NTP budget for MDR-TB, while domestic funding comprised 19%, leaving a 55% funding gap. Based on annual data reported to WHO, MDR-TB patient enrolment in treatment sites previously supported by the MDR-TB grant declined nationwide by 70% in the two quarters following the closure of the Global Fund. These challenges combined with the need to improve treatment success rates point to issues that could jeopardise the investments in MDR-TB control in China.

China is developing strategies and policies to address these challenges. During NCE, earmarked funds for medical services were replaced with a financing mechanism through which the Global Fund and local authorities shared costs via health insurance schemes. Further, a model for sustainable financial and care packages for TB and MDR-TB patients conducted in four prefectures was also piloted [22]. Additional policies to ensure the provision of a MDR-TB quality package of care and protection of patients from medical and associated non-medical costs include: increased insurance reimbursement rates (up to 70%), social protection funds for the poorest patients and the continued use of the existing public health package for TB. Also, case-based payment is being introduced as a measure to control hospital costs, and ensure quality of delivery. Selected provinces have further legislated financial protective measures to avoid catastrophic expenditure and harmonise reimbursement rates across the different health system levels.

China is moving into a new era marked by full country ownership and financing of MDR-TB services. The country’s health system has inherited a formidable legacy from the achievements of the partnership with the Global Fund, including strengthened capacity in terms of infrastructure, technology, and human resources, at all levels of a pre-existing network. The collaboration has also helped identify potential solutions to existing gaps in service delivery and financial coverage.

China’s commitment to basic TB control is the foundation of a broad strategy to prevent drug-resistance before it emerges. A strong public health programme aimed at eliminating TB through an effective package is a public health good, for China and the world. China’s success in controlling drug-sensitive TB, its scale-up of MDR-TB diagnostic and treatment capacities, and the graduation from the Global Fund serves as an important example for other countries. Going forward, as TB control and care shift to hospitals, new policies must draw on programmatic research for the timely response to challenges as they arise.

At the same time, China is at a critical juncture, whereby TB and MDR-TB control will hold a mirror to the effectiveness of hospitals’ greater public health role. With the end of the Global Fund project, health care reforms mark a turning point in resource mobilization towards country self-sufficiency and a comprehensive delivery system to ensure quality of care, providing an exceptional opportunity to show, 20 years from now, how reforms proved pivotal to the elimination of TB, one of China’s most important public health challenges due to an infectious disease.

## Supporting information

S1 Fig TB cases tested for MDR-TB (new and retreatment combined), by province and year. (TIF)

S2 Fig Correlation between sputum smear and culture conversion at 6 months for MDR-TB patients on treatment, by province, October 2006–January 2014. Each bubble represents one province, with the size of the bubble proportional to the log of the number of cases fitted with linear smooth line and 95% s.e. Dotted line = unity.
(TIF)

S1 Table Provincial and regional breakdown of treatment success rate. ^a^ Eastern Provinces: Beijing, Fujian, Guangdong, Guangxi, Hainan, Hebei, Jiangsu, Liaoning, Shandong, Shanghai, Tianjin, and Zhejiang.
^b^ Central Provinces: Anhui, Heilongjiang, Henan, Hubei, Hunan, Inner Mongolia, Jiangxi, Jilin, and Shanxi.
^c^ Western Provinces: Chongqing, Gansu, Guizhou, Ningxia, Qinghai, Shaanxi, Sichuan, Xinjiang, Yunnan, and Xizang.
(DOCX)
