# Supplementary figures and images for: The Global Fund in China: Multidrug-resistant tuberculosis nationwide programmatic scale-up and challenges to transition to full country ownership
Source: PLoS One. 2017 Jun 19;12(6):e0177536. doi: 10.1371/journal.pone.0177536 (PMC5476250; doi:10.1371/journal.pone.0177536)

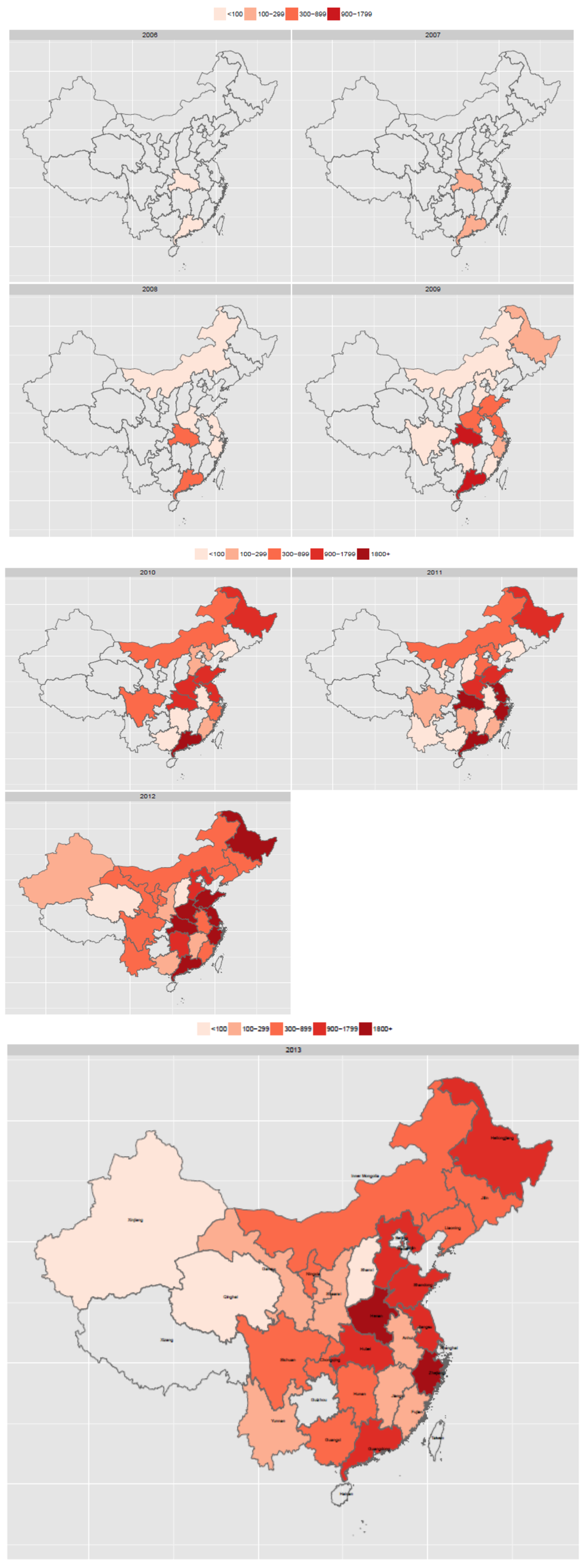

Supplement: S1 Fig — (TIF) [file pone.0177536.s001.tif]

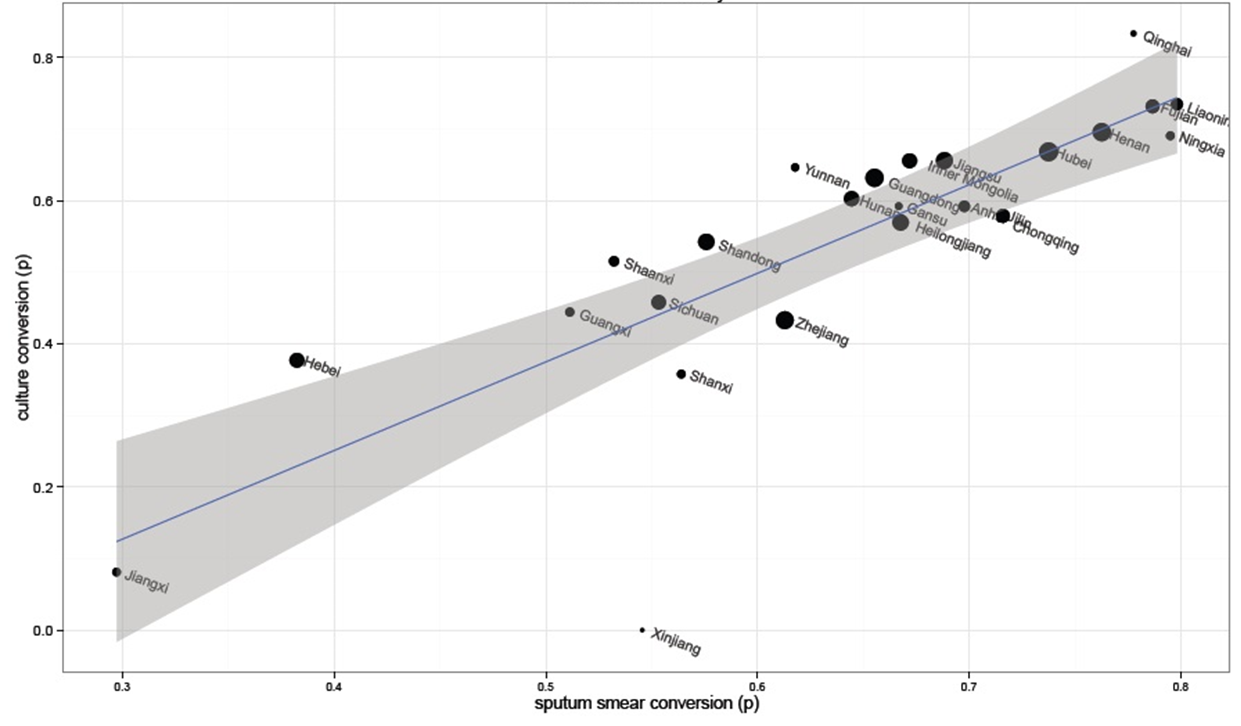

Supplement: S2 Fig — Each bubble represents one province, with the size of the bubble proportional to the log of the number of cases fitted with linear smooth line and 95% s.e. Dotted line = unity. (TIF) [file pone.0177536.s002.tif]
