# Supplementary material for: The Global Fund in China: Multidrug-resistant tuberculosis nationwide programmatic scale-up and challenges to transition to full country ownership
Source: PLoS One. 2017 Jun 19;12(6):e0177536. doi: 10.1371/journal.pone.0177536 (PMC5476250; doi:10.1371/journal.pone.0177536)
Supplement: S1 Table — a Eastern Provinces: Beijing, Fujian, Guangdong, Guangxi, Hainan, Hebei, Jiangsu, Liaoning, Shandong, Shanghai, Tianjin, and Zhejiang. b Central Provinces: Anhui, Heilongjiang, Henan, Hubei, Hunan, Inner Mongolia, Jiangxi, Jilin, and Shanxi. c Western Provinces: Chongqing, Gansu, Guizhou, Ningxia, Qinghai, Shaanxi, Sichuan, Xinjiang, Yunnan, and Xizang. (DOCX) [file pone.0177536.s003.docx]

|  | **Number of patients with available treatment outcomes** | **Treatment success rate**  **(%)** |
| --- | --- | --- |
| **Eastern Provinces^a^** | | |
| Fujian | 91 | 56.0 |
| Guangdong | 415 | 52.5 |
| Hebei | 58 | 39.7 |
| Jiangsu | 269 | 51.7 |
| Shandong | 285 | 38.6 |
| Zhejiang | 331 | 45.9 |
| **Total** | **1,449** | **47.8** |
| **Central Provinces^b^** | | |
| Heilongjiang | 164 | 55.5 |
| Henan | 325 | 49.2 |
| Hubei | 511 | 54.9 |
| Hunan | 57 | 35.1 |
| Inner Mongolia | 152 | 47.4 |
| **Total** | **1,209** | **51.6** |
| **Western Provinces^c^** | | |
| **Sichuan** | 134 | 24.6 |
| **National** | | |
| **Total** | **2,792** | **48.4** |
